# Live attenuated pertussis vaccine for prevention and treatment of allergic airway inflammation in mice

**DOI:** 10.1038/s41541-022-00494-w

**Published:** 2022-06-23

**Authors:** Thomas Belcher, Saliha Ait-Yahia, Luis Solans, Anne-Sophie Debrie, Stephane Cauchi, Anne Tsicopoulos, Camille Locht

**Affiliations:** 1 Univ. Lille, CNRS, Inserm, CHU Lille, Institut Pasteur de Lille, U1019-UMR9017-CIIL-Centre d’Infection et d’Immunité de Lille, Lille, France; 2 Present Address: Luis Solans, Exopol, Poligono Rio Gallego, San Mateo de Gallego, Spain

**Keywords:** Live attenuated vaccines, Microbiology

## Abstract

Live attenuated vaccines often have beneficial non-specific effects, protecting against heterologous infectious and non-infectious diseases. We have developed a live attenuated pertussis vaccine, named BPZE1, currently in advanced clinical development. Here, we examined the prophylactic and therapeutic potential of its pertactin-deficient derivative BPZE1P in a mouse model of house dust mite (HDM)-induced allergic airway inflammation (AAI). BPZE1P was given nasally either before or after sensitization with HDM, followed by HDM challenge, or between two challenge episodes. Vaccination prior to sensitization reduced resistance in the airways, the numbers of infiltrating eosinophils and the concentrations of proinflammatory cytokines, such as IL-1α, IL-1β and IL-33, in the lungs but had no effect on Th2 cytokine levels. BPZE1P also protected when delivered after sensitization or between two challenge episodes. However, in this case the levels of Th2 cytokines in the lung were decreased without significant effects on IL-1α, IL-1β and IL-33 production. The vaccine restored lung function and decreased eosinophil influx in the lungs of HDM-treated mice. BPZE1P has a better take than BPZE1 in hosts vaccinated with acellular pertussis vaccines. Therefore, it has interesting potential as a preventive and therapeutic agent against AAI, even in acellular pertussis-vaccinated populations.

## Introduction

In addition to inducing antigen-specific immune responses for which most vaccines are initially designed, evidence has accumulated over the years indicating that some vaccines may also have protective effects against diseases for which they were not initially intended. These non-specific effects, also referred to as off-target effects, have been well documented for live vaccines. As such, the live attenuated anti-tuberculosis vaccine Bacillus Calmette-Guérin (BCG) was shown decades ago to decrease childhood mortality far beyond what would be expected merely by protection against tuberculosis^1^. Especially heterologous protection against respiratory infections and sepsis has been well documented^2^, but there is also evidence from nonclinical and clinical studies that BCG vaccination may be associated with a favorable outcome of noninfectious, autoimmune and inflammatory diseases^3^, including allergic asthma^4^. Beneficial off-target effects have also been described for other live vaccines, such as measles^5^ and oral polio vaccines^6^. In contrast to live vaccines, non-live vaccines, such as the diphtheria-tetanus-pertussis vaccines, have been suggested to be associated with increased childhood mortality^7^.

We have developed a live attenuated nasal pertussis vaccine, named BPZE1, which is based on the genetic removal or detoxification of the three major *Bordetella pertussis* toxins: dermonecrotic toxin, tracheal cytotoxin and pertussis toxin^8^. The vaccine has been shown to be safe and immunogenic in several animal models (for review see^9^), as well as in humans during several clinical trials^10,11^.

Previous studies have shown that, in addition of being highly protective against pertussis in mice^12^ and nonhuman primates^13^, BPZE1 also provides protection against non-related diseases^14^. In this study we used a mouse model to examine the effect of a pertactin-deficient BPZE1 derivative, named BPZE1P^15^, on the prevention and treatment of house dust mite (HDM)-induced allergic airway inflammation (AAI). Previous studies in a mouse model of ovalbumin (OVA)-induced AAI have shown that BPZE1 administration prior to allergen sensitization reduces airway eosinophil influx and Th2 cytokine responses in the lungs^16,17^. Here, we extend these initial observations using BPZE1P in the more relevant HDM model and show that nasal administration of BPZE1P either prior to or after HDM sensitization, as well as between two challenge episodes decreases AAI and improves lung function. The pertactin-deficient BPZE1P was used, as pertactin is a protective antigen present in most current pertussis vaccines, and antibodies to pertactin have been shown to hamper BPZE1 vaccine take in both mice^15^ and humans^10^.

## Results

### BPZE1P and BPZE1 protect equally well in a prophylactic model against HDM-induced AAI

**Fig. 1:**
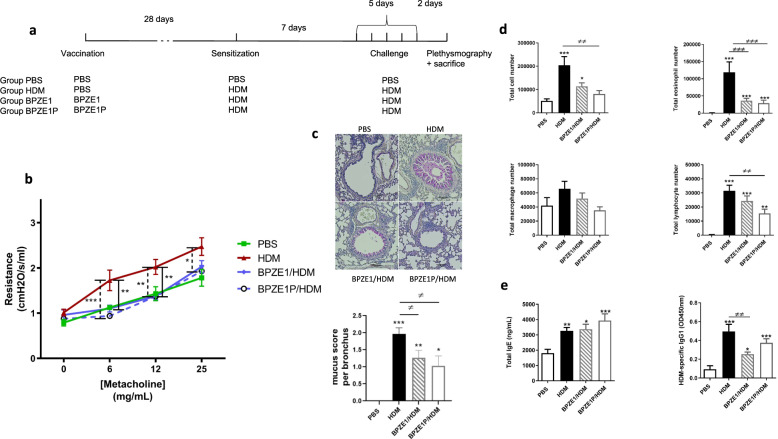
Prophylactic protection by BPZE1 or BPZE1P against HDM-induced AAI. **a** Outline of experimental design. Mice were vaccinated once with BPZE1 or BPZE1P (BPZE1/HDM and BPZE1P/HDM). Two other groups were left unvaccinated (PBS and HDM). 28 days later the vaccinated groups plus one unvaccinated group (HDM) were sensitized with HDM and challenged 7 days later with HDM daily for 5 days. Two days after the final challenge, plethysmography was performed and serum, BAL and whole lungs were collected. **b** Airway resistance to increasing concentrations of nebulised methacholine (PBS *n* = 6; HDM, BPZE1/HDM and BPZE1P/HDM *n* = 7–8). **c** Representative microphotographs of PAS-stained lung sections and quantitative evaluation of mucus. Mucus is stained in pink and inflammatory cells in blue. Scale bars: 100 μm. Data measurements were performed on 6 different lung sections from 2 mice per group. **d** Total cell, eosinophil, macrophage and lymphocyte numbers (PBS *n* = 7; HDM, BPZE1/HDM and BPZE1P/HDM *n* = 12–14). Data are representative of 2 independent experiments. **e** Total serum IgE and HDM specific IgG1 levels (PBS *n* = 7–8; HDM *n* = 13; HDM/BPZE1 and HDM/BPZE1P *n* = 15–16). Data are representative of 2 independent experiments and represent the mean values, with SEM. */≠, *p* < 0.05; **/≠≠, *p* < 0.01; ***/≠≠≠, *p* < 0.001. For resistance **b** * indicates significance between HDM and BPZE1/HDM (solid line) or BPZE1P/HDM (dashed line). For cell counts **d** and Ig levels **e** * indicates significance compared to PBS group and ≠ indicates significance between the groups indicated. Two-way repeated measure analysis of variance with Bonferroni post-test for resistance, Kruskal-Wallis with Conover post-test for Ig levels, mucus score and cell counts.

As pertactin has been associated with immunomodulatory effects of *B. pertussis*^18^, we first examined whether the deletion of the pertactin gene in BPZE1P might affect the protective effect of the vaccine against AAI. In a prophylactic model of HDM-induced AAI we therefore compared the protective effect of BPZE1P with that of BPZE1. Mice were vaccinated with 10^6^ CFU BPZE1 or BPZE1P and sensitized with 5 IR HDM extract 28 days later, followed by HDM challenge 7 days after sensitization (Fig. 1a). Control groups were not vaccinated but sensitized and challenged (HDM group), or remained untreated (PBS group). Following challenge, the resistance of the airways to increasing concentrations of methacholine was measured by plethysmography. The HDM group displayed significantly higher levels of resistance than the PBS group (Fig. 1b). Both the BPZE1 and BPZE1P groups showed significantly decreased levels of airway resistance compared to the HDM group. The two vaccinated groups were not significantly different from the PBS group, and there was no difference between BPZE1- and BPZE1P-vaccinated mice.

Histological analyses of lung sections from HDM-challenged mice showed that the increase in Periodic acid Schiff (PAS)-stained mucus (pink color) in HDM-sensitised and -challenged mice was decreased in mice vaccinated by BPZE1 or BPZE1P compared to those that were not vaccinated, and there was no significant difference between the two vaccinated groups (Fig. 1c). Similarly, one vaccination with either BPZE1 or BPZE1P was sufficient to reduce the level of peri-bronchial inflammation (cell infiltration in blue) (Fig. 1c).

Consistent with previous studies^19^, the HDM group displayed increased total cell numbers compared to the PBS group, as well as elevated eosinophil and lymphocyte levels in the bronchoalveolar lavage (BAL) (Fig. 1d). Mice vaccinated with BPZE1 or BPZE1P displayed lower levels of total cells and eosinophils compared to the HDM group (Fig. 1d), with no significant difference between the two vaccinated groups. The decrease in lymphocyte numbers in the BAL of vaccinated mice compared to the HDM group was less pronounced and significant only for BPZE1P, while there was no significant difference in macrophage numbers between any of the groups (Fig. 1d).

Total serum IgE levels were elevated for the HDM group compared to the PBS group (Fig. 1e). Vaccination with BPZE1 or BPZE1P did not reduce the total serum IgE levels (Fig. 1e). Since HDM-specific IgE were under the limits of detection in our assays (data not shown), we chose to analyze specific IgG1 levels as a representative of Th2 humoral responses. HDM-specific serum IgG1 levels were increased in the HDM group compared to the PBS group, but they were decreased in the BPZE1 and BPZE1P groups compared to the HDM group, although this decrease did not reach statistical significance for the BPZE1P group (Fig. 1e).

Together these results indicate that BPZE1 and BPZE1P protect equally well against HDM-induced AAI and thus that the absence of pertactin does not affect the protective properties of the live vaccine.

### BPZE1P vaccination reduces lung inflammatory cytokine response to HDM challenge

**Fig. 2:**
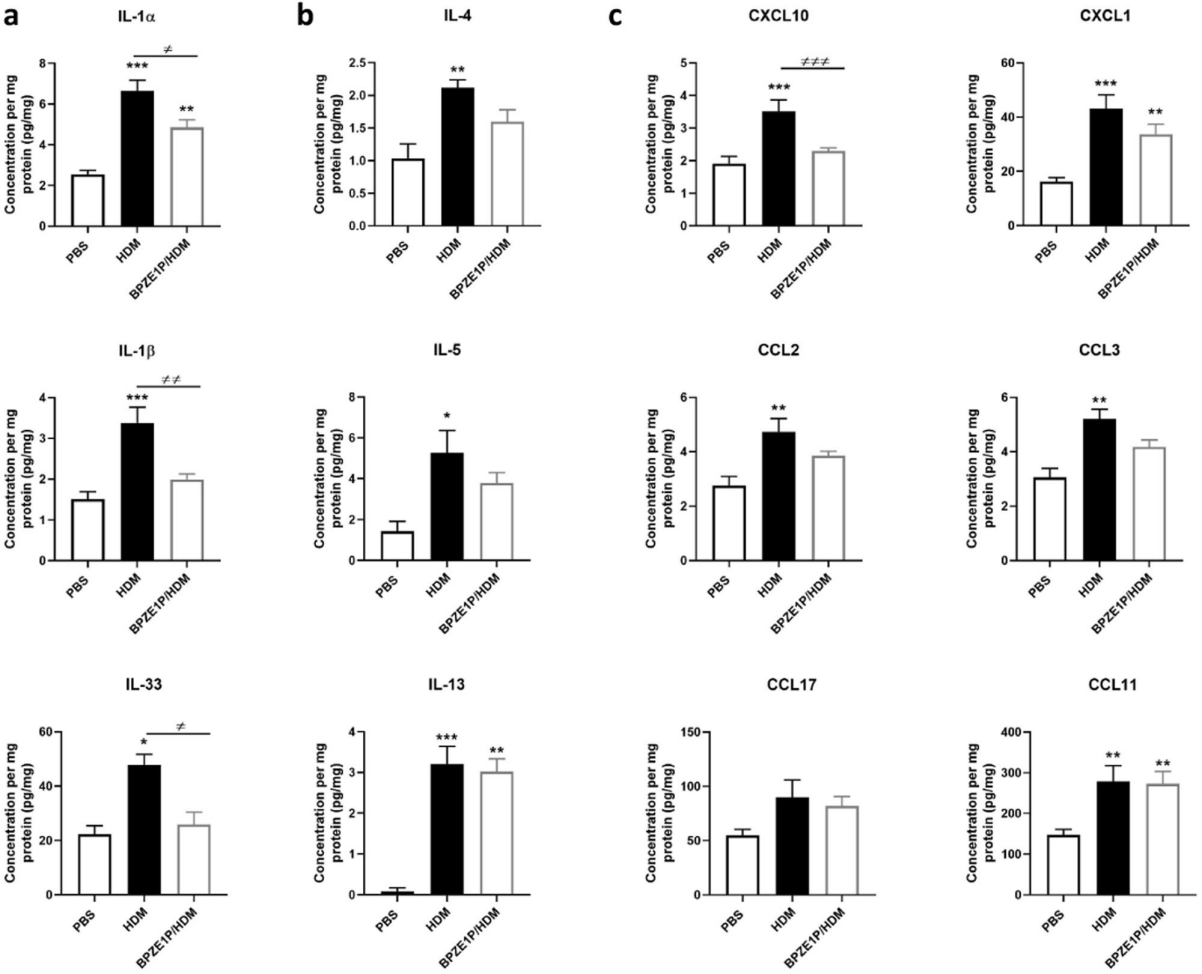
Cytokine and chemokine concentrations in the lungs normalized to concentration of total protein. Cytokine and chemokine levels in lung extracts were measured by Luminex or ELISA. Data represent the mean values with SEM. PBS *n* = 3–6; HDM and BPZE1P *n* = 6–8. */≠, *p* < 0.05; **/≠≠, *p* < 0.01. ***/≠≠≠, *p* < 0.001. * indicates significance compared to PBS group, ≠ indicates significance compared to groups indicated, Kruskal-Wallis with Conover post-test.

Since BPZE1 and BPZE1P protected equally well in the prophylactic model of HDM-induced AAI, we concentrated cytokine/chemokine analyses on the BPZE1P group in comparison with the HDM and PBS groups. We analyzed the levels of IL-1α, IL-1β, IL-2, IL-4, IL-5, IL-6, IL-9, IL-10, IL-12, IL-13, IL-17, IL-18, IL-22, IL-23, IL-27, IL-33, IFN-β, TNF-α, IFN-γ, GM-CSF, CXCL1, CXCL2, CXCL9, CXCL10, CCL2, CCL3, CCL4, CCL5, CCL7, CCL11, CCL17, VEGF, FGF-β in lung extracts. We chose lung extracts rather than BAL as used in previous studies^17^, because of higher concentrations detected in lung extracts compared to BAL. While for most, there was no difference between the groups (data not shown), increased levels of several cytokines/chemokines were measured in lung extracts from the HDM group compared to the PBS group (Fig. 2a–c). The levels of IL-1α, IL-1β, and IL-33, essentially produced by innate immune cells and epithelial cells, were significantly decreased in the BPZE1P group compared to the HDM group (Fig. 2a). In contrast, there was only a trend without reaching significance in the decrease of Th2 cytokine levels, mainly induced by lymphocytes, between the HDM and the BPZE1P groups (Fig. 2b). CXCL10 levels were significantly lower in the BPZE1P group compared to the HDM group (Fig. 2c), while there were trends for the decrease of other chemokines, such as CCL2, CXCL1 and CCL3. CCL17 and CCL11 levels in the BPZE1P group were not different from the HDM group, but CCL17 was barely increased in the HDM group compared to the PBS group.

### BPZE1P protects against HDM-induced AAI when administered after sensitization

**Fig. 3:**
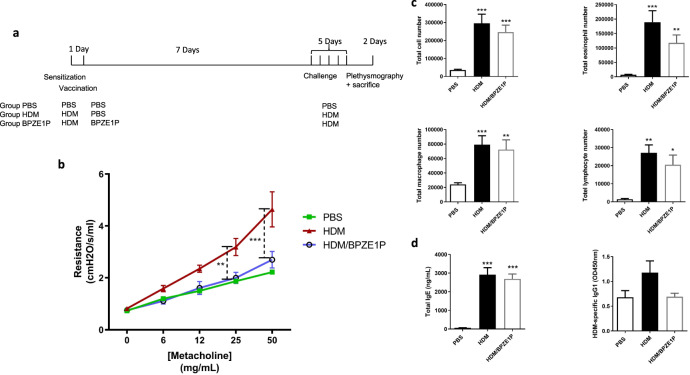
Therapeutic effects BPZE1P in mice in a short model of HDM-induced AAI. **a** Outline of experimental design. Two groups of mice were sensitized with HDM, while a third group was not. One day later one of the sensitized groups was vaccinated with BPZE1P (HDM/BPZE1P). Seven days later both sensitized groups that were challenged with HDM daily for 5 days. The PBS group had been neither vaccinated, sensitized nor challenged. Two days after the final challenge, plethysmography was performed and serum, BAL fluid and whole lungs were collected. **b** Airway resistance to increasing concentrations of nebulised methacholine. All groups *n* = 7. **c** Total cell numbers in the BAL and numbers of eosinophils, lymphocytes and macrophages (PBS *n* = 6; HDM, and HDM/BPZE1P *n* = 7–8). **d** Total serum IgE and HDM-specific IgG1 levels. IgE: PBS *n* = 8; HDM and HDM/BPZE1P *n* = 10. IgG1: PBS *n* = 3; HDM *n* = 8; HDM/BPZE1P *n* = 10. Data represent the mean values, with SEM. */≠, *p* < 0.05; **/≠≠, *p* < 0.01; ***/≠≠≠ *p* < 0.001; for resistance * indicates significance between HDM and HDM/BPZE1P (dashed line), for cell counts and Ig levels * indicates significance compared to PBS group and ≠ indicates significance between the groups indicated, Kruskal-Wallis with Conover post-test for Ig levels and cell counts, two-way repeated measure analysis of variance with Bonferroni post-test for resistance.

To determine whether BPZE1P vaccination offers protection against HDM-induced AAI when administered after HDM sensitization, mice were inoculated with BPZE1P one day after sensitization with 5 IR of HDM. HDM challenge was carried out 7 days after inoculation by intranasal administration of HDM daily for 5 days, and animals were sacrificed 2 days later (Fig. 3a). Resistance in the lungs was measured by plethysmography, and cell infiltration in the BAL was quantified, as well as serum IgE and HDM-specific IgG1 levels. Administration of BPZE1P one day after sensitization significantly decreased resistance in the airways compared to the HDM group (Fig. 3b). Eosinophil numbers in the BAL were also reduced in the BPZE1P group compared to the HDM group, although the difference did not reach statistical significance (Fig. 3c). There was no effect of BPZE1P vaccination on the numbers of macrophages and total lymphocytes in the BAL. This may be due to the fact that BPZE1P likely continues to be present in the lung^8^ at the time of challenge and analyses, and bacteria, presumed to be BPZE1P, were indeed observed in the BAL of sacrificed animals (not shown). Finally, while there was no difference in the levels of total serum IgE of the vaccinated groups versus the HDM group, there was a trend towards a decrease in levels of HDM-specific IgG1 (Fig. 3d).

### BPZE1P protects against HDM-induced AAI when administered between two HDM challenges

**Fig. 4:**
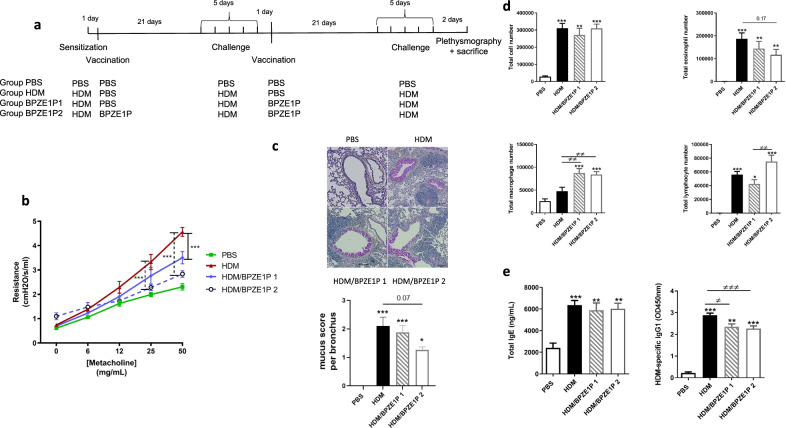
Therapeutic protection by BPZE1P in a mouse model of HDM-induced AAI. **a** Outline of experimental design. 3 groups of mice were sensitized to HDM while a fourth group was not. One sensitized group was vaccinated once with BPZE1P one day after sensitization (HDM/BPZE1P 2). 28 days later the three sensitized groups were challenged with HDM daily for 5 days. One day after the challenge the HDM/BPZE1P 2 group received a second dose of BPZE1P, while another one of the 3 sensitized and challenged groups received a first dose of BPZE1P (HDM/BPZE1P 1). 28 days later the three sensitized and challenged groups were challenged again daily for 5 days. The remaining unvaccinated group (PBS) was neither sensitized nor challenged. Two days after the final challenge, plethysmography was performed and serum, BAL fluid and whole lungs were collected. **b** Airway resistance to increasing concentrations of nebulised methacholine. PBS *n* = 6; HDM, HDM/BPZE1P 1 and HDM/BPZE1P 2 *n* = 7–8. **c** Representative microphotographs of PAS-stained lung sections and quantitative evaluation of mucus. Mucus is stained in pink and inflammatory cells in blue. Scale bars: 100 μm. Data measurements were performed on 6 different lung sections from 2 mice per group. **d** Total cell numbers in the BAL and numbers of eosinophils, lymphocytes and macrophages. PBS *n* = 6–7; HDM, HDM/BPZE1P 1 and HDM/BPZE1P 2 *n* = 13–14. Data are representative of 2 independent experiments. **e** Total serum IgE and HDM-specific IgG1 levels. Data are representative of 2 independent experiments. IgE: PBS *n* = 7; HDM, HDM/BPZE1P 1 and HDM/BPZE1P 2 *n* = 14–16. IgG1: PBS *n* = 7; HDM *n* = 16; HDM/BPZE1P 1 *n* = 6; HDM/BPZE1P 2 *n* = 14. Data represent the mean values, with SEM. */≠, *p* < 0.05; **/≠≠, *p* < 0.01; ***/≠≠≠, *p* < 0.001; ns, not significant. For resistance * indicates significance between HDM and HDM/BPZE1P 1 (solid line) or HDM/BPZE1P 2 (dashed line). For cell counts and Ig levels * indicates significance compared to PBS group and ≠ indicates significance between the groups indicated. Two-way repeated measure analysis of variance with Bonferroni post-test for resistance, Kruskal-Wallis with Conover post-test for Ig levels, mucus score and cell counts.

In order to not confound the immune response to HDM allergen by that of bacteria that are still present we switched to a long-term model in which HDM challenge was performed three weeks after BPZE1P administration. In this model mice were sensitized with 25 IR HDM and 3 weeks later challenged daily for five consecutive days. After 3 weeks, the mice were again challenged daily with HDM for five consecutive days. One group was vaccinated one day after the initial sensitization and a second time one day after the initial round of HDM challenge (BPZE1P 2). The second group was vaccinated only once after the initial round of HDM challenge (BPZE1P 1). The two additional groups were not vaccinated but sensitized and challenged (HDM), or left untreated (PBS) (Fig. 4a).

BPZE1P vaccination once after one round of HDM challenge significantly decreased airway resistance compared to the HDM group (Fig. 4b). Airway resistance was further decreased in the BPZE1P 2 group. Histological analyses showed that while in the BPZE1P 1 group there was no significant reduction in PAS-stained mucus compared to the non-vaccinated mice, a trend of decreased mucus secretion was seen in the BPZE1P 2 group, compared with the HDM group, which did not quite reach statistical significance. There was no variation of the cell infiltration (Fig. 4c). Total cell counts in the BAL were unaffected by BPZE1P, while macrophage numbers were increased for the two vaccinated groups (Fig. 4d). There was a trend to a reduction in the number of eosinophils in the BAL in the BPZE1P 1 group, and a further decrease in the BPZE1P 2 group (Fig. 4d). Finally, while there was no difference in the numbers of lymphocytes in the BAL when one dose of BPZE1P was administered, an increase was seen in the group that received two doses (Fig. 4d).

No decrease in total serum IgE levels was observed when BPZE1P was given, but there was a significant reduction in levels of HDM-specific serum IgG1, a difference which was most significant for the BPZE1P 2 group (Fig. 4e).

**Fig. 5:**
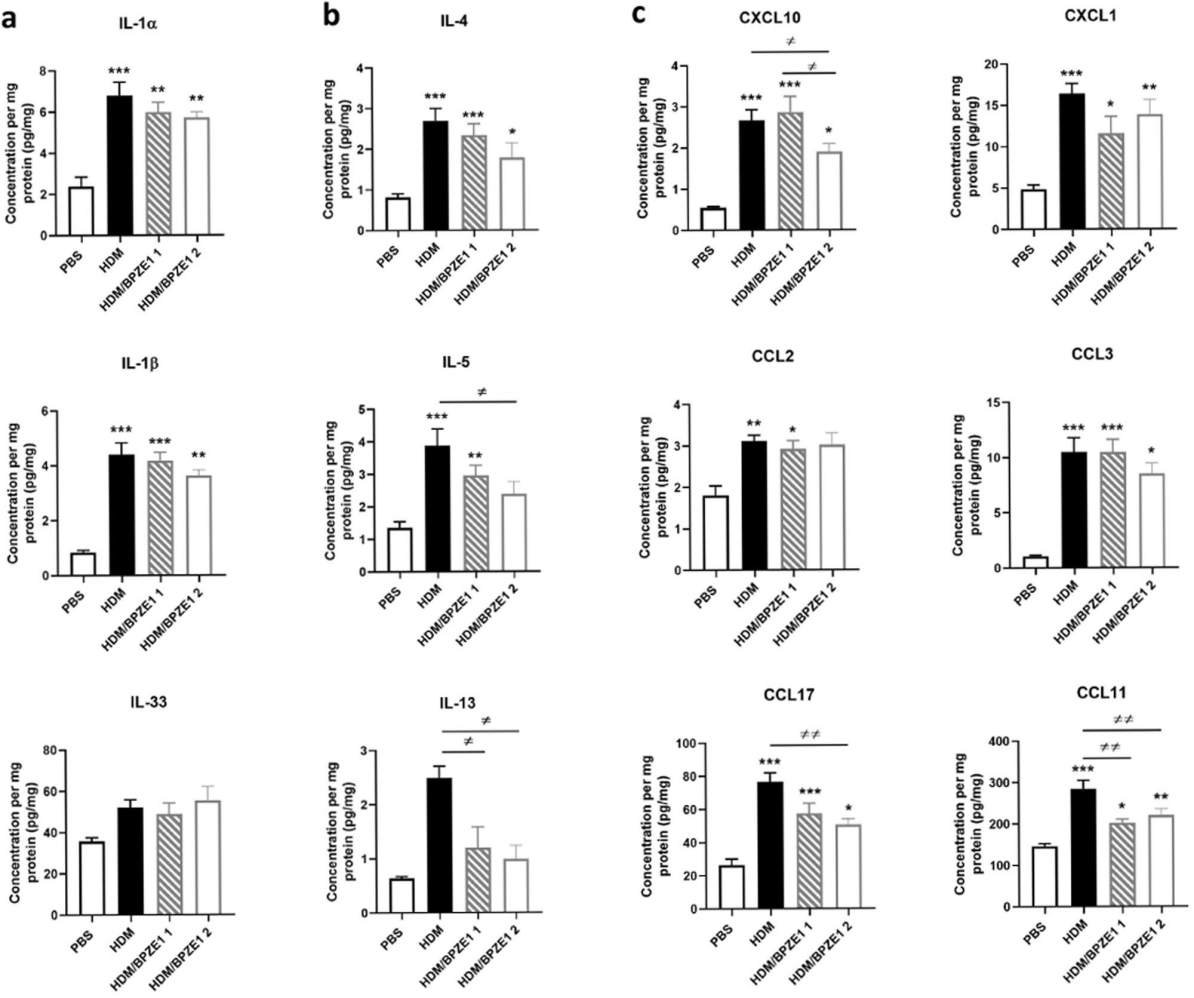
Therapeutic effects of BPZE1P on cytokine and chemokine profiles of the lungs from mice in a model of HDM-induced AAI. Cytokine and chemokine levels in lung extracts were measured by Luminex and normalized to total protein concentration. Data represent the mean values with SEM. PBS *n* = 6–7; HDM, HDM/BPZE1 1 and BPZE1P 2 *n* = 6–10. */≠, *p* < 0.05; **/≠≠, *p* < 0.01; ***/≠≠≠, *p* < 0.001; * indicates significance compared to PBS group, ≠ indicates significance compared to groups indicated, Kruskal-Wallis with Conover post-test.

In contrast to the prophylactic model described above, BPZE1P vaccination did not reduce the levels of IL-1α, IL-1β or IL-33 compared to the HDM group (Fig. 5a). Instead, it significantly decreased the levels of Th2 cytokines IL-5 and IL-13 compared to the HDM group, which was most striking for the BPZE1P 2 group (Fig. 5b). There was also a trend towards a decrease in the IL-4 levels in the BPZE1P 2 group compared to the HDM group (Fig. 5b). Furthermore, we observed significant decreases in the BPZE1P 2 group compared to the HDM group for CXCL10 and CCL17. Decreases in CCL11 levels were significant for both vaccinated groups (Fig. 5c). No significant decrease in CXCL1, CCL2 and CCL3 levels were observed for the vaccinated groups compared to the HDM group.

## Discussion

Allergic asthma is a severe chronic respiratory disease which affects over 330 million people worldwide^20^, with increasing incidence among young children^21^. The disease is characterised by airway hyperresponsiveness with obstruction of the airways, driven in part by Th2 responses and high IgE levels^22^. Current treatments rely mostly on inhaled corticoids and, in some severe forms, on biotherapies targeting IgE or Th2 cytokines and their receptors. However, these treatments have met with variable success^23^, and alternative or adjunct treatment of AAI patients are urgently needed.

Through their non-specific effects, some live vaccines may be useful in the management of AAI^4^. In contrast to infection by virulent *B. pertussis*, which exacerbates disease in mouse models of AAI^24,25^, and evidence in humans showing that contracting whooping cough is a risk factor for asthma development^26,27^, we show here that nasal administration of the live attenuated *B. pertussis* strain BPZE1P prior to or after HDM sensitization protects against rather than exacerbates AAI in mice. In addition to a decrease in eosinophil influx into the lungs BPZE1P vaccination also restored lung function, as airway resistance upon methacholine administration was significantly reduced in BPZE1P-vaccinated mice after HDM challenge compared to the non-vaccinated mice and reached that of control mice.

Pertactin is a major antigen of acellular pertussis vaccines currently used in most industrialized countries, and we have previously shown in a phase 1 clinical trial that BPZE1 vaccine take was hampered by pre-existing anti-pertactin antibodies^10^. Considering the extensive use of pertactin-containing pertussis vaccines^28,29^, and the fact that in current acellular pertussis vaccines pertactin is the only antigen inducing bactericidal antibodies^30^, anti-pertactin antibodies induced by these vaccines may thus limit the potential use of BPZE1 for the treatment of AAI. We therefore tested the pertactin-deficient BPZE1 derivative BPZE1P instead of BPZE1, used in previous studies^16,17^, to allow for optimal colonization and anti-AAI effects in acellular pertussis vaccinated subjects. However, pertactin has been shown to have immunomodulatory properties, and strains lacking pertactin induce a greater production of pro-inflammatory cytokines from monocyte-derived dendritic cells in vitro than strains producing pertactin^18^. The deletion of the pertactin gene in BPZE1P may therefore potentially alter its protective potential against AAI. However, by comparing BPZE1 with BPZE1P in the model in which the vaccines were administered before HDM sensitization, we found that both strains protected equally well. The decrease in methacholine-induced airway resistance and eosinophil influx was indistinguishable between the two strains, making BPZE1P an interesting candidate for the prevention of AAI.

While both strains reduced eosinophil influx in the lungs and HDM-specific serum IgG1, compared to non-vaccinated mice, neither reduced the induction of total serum IgE, a finding different from what was described for the OVA-induced AAI model^17^. However, HDM induced much higher levels of total IgE (approximately 3,000 ng/ml) than OVA (approximately 150 ng/ml)^17^, and the vaccine doses given in this study were five-fold lower than in the study by Li et al. It is therefore possible that the levels of IgE induced by HDM were too high to be down-regulated by a relatively low dose of BPZE1 or BPZE1P.

BPZE1P vaccination prior to sensitization significantly reduced IL-1α, IL-1β and IL-33 production in the lungs of HDM-treated mice compared to the non-vaccinated mice, whereas the production or IL-4 and IL-5 was only modestly decreased, and no decrease was seen for IL-13. Decreased levels of IL-5 and IL-13 have, however, been seen in the BAL of mice sensitized and challenged with OVA after BPZE1 vaccination^16,17^. Several reasons may potentially account for these differences. In this study, we analyzed cytokine concentrations in total lung extracts, while in the previous study BAL were analyzed. Furthermore, sensitization with OVA was performed by intraperitoneal challenge, while here sensitization by HDM was intranasal. Most importantly, as mentioned above, a five-fold lower dose of vaccine used in this study, compared to one of the previous studies^17^, while in the other previous study^16^ OVA sensitization was performed at the peak of infection (day 10), instead of HDM sensitization performed here 28 days after BPZE1 administration, when the bacteria had cleared from the lung.

IL-1α and IL-1β are mostly produced by innate immune cells, such as monocytes and macrophages, which leads to IL-33 release by epithelial cells, thereby activating dendritic cells to favour a Th2 response^31^. The three cytokines have been shown to participate in the sensitization process to HDM and can act directly on structural cells, such as mucus cells, smooth muscle cells and fibroblasts and thereby contribute to airway remodelling and alterations of lung function^32–34^. These cytokines are considered “upstream” cytokines driving Th2 responses in the context of asthmatic disease^35,36^. The down-regulation of IL-33, IL-1α and IL-1β production when BPZE1P was given before HDM sensitization may thus underlie the immune mechanism of BPZE1P-mediated protection against airway resistance and mucus production in a prophylactic setting, even without a decrease in Th2 cytokine production.

BPZE1P also protected against AAI when given after HDM sensitization or between two rounds of HDM challenge in a therapeutic setting. In both cases, BPZE1P rescued lung function, resulted in the reduction of eosinophil influx and HDM-specific IgG1 production, but had no effect on total IgE production. Interestingly, when BPZE1P was administered between two challenge rounds the cytokine profiles in the lungs were different from those seen when BPZE1P was given before HDM sensitization. In the post-sensitization setting the levels of IL-33, IL-1α and IL-1β in the lungs were not reduced when compared to the non-vaccinated mice, as these sensitizing cytokines had already been induced prior to BPZE1P administration. Instead, the levels of Th2 cytokines were significantly decreased when BPZE1P was given between challenges, especially after two doses of BPZE1, thereby modifying the effector phase of AAI. The mechanism for the modulation of AAI by BPZE1P is thus different between the prophylactic and the therapeutic model. When given prophylactically (i.e. pre-sensitization), BPZE1P may have an effect on the ability of first line cells in the lungs, such as epithelial cells, to secrete IL-33 in response to later HDM sensitization and challenge. In contrast, when given therapeutically (post-sensitization), BPZE1 may not affect the levels of IL-33, but act on the effector mechanisms of AAI by modulating the levels of Th2 cytokines. Thus, BPZE1P acts in a dual manner to modulate AAI.

Levels of CXCL10 in lungs of mice vaccinated with BPZE1P were decreased in both the prophylactic model and the therapeutic model. This chemokine is generally considered a pro-Th1 chemokine. However, in a model of AAI it has been shown that its overexpression promotes airway hyperresponsiveness and eosinophilia, while both were decreased in its absence^37^. Eosinophils express the CXCL10 receptor and are activated by this chemokine^38^, explaining why eosinophil numbers were decreased in the prophylactic model even in the absence of a BPZE1P effect on IL-5 and CCL11 expression. Furthermore, trends towards a decrease in levels of CXCL1, CCL2 and CCL3 were observed in the prophylactic model but were not apparent in the therapeutic model. On the other hand, in the therapeutic model, levels of CCL17 and CCL11 were significantly decreased in vaccinated mice, which for CCL17 was only significant for mice which had received two doses, again revealing the boosting effect of the vaccine in this model. These chemokines attract Th2 cells^39^ and eosinophils^40^ through CCR4 and CCR3, respectively, which may explain the BPZE1P effect on eosinophils and Th2 cells in the therapeutic model. The different effects on chemokine levels between the two models thus confirm that different mechanisms for the protective action of BPZE1P are at play, depending on the model.

In the post-sensitization setting, we compared two groups of BPZE1P-treated mice. BPZE1P was either given once between two HDM challenges, or given once before the first challenge and a second time in between the two challenge episodes. Although a single BPZE1P administration between the two challenge rounds already significantly decreased AAI, two BPZE1P administrations showed a further modulated phenotype, when the airway resistance and number of infiltrating eosinophils were examined, indicating that BPZE1P has a booster effect in this model of AAI. This booster effect was also seen when chemokine production, in particular CXCL10 and CCL17, was examined. A booster effect of BPZE1 has been shown previously in a mouse model of influenza^41^, in a model of contact dermatitis^17^, and more recently in a model of invasive pneumococcal disease^42^.

In summary, we report here that BPZE1P has off-target effects similar to BPZE1 in a mouse model of HDM-induced AAI. We also show the ability of BPZE1P to protect in therapeutic models when given between sensitization and challenge or between two challenge episodes. The lung cytokine profiles suggest that in the prophylactic setting BPZE1P exerts is protective effect by reducing the HDM-induced production of pro-inflammatory and upstream cytokines, such as IL-1α, IL-1β and IL-33, while in the therapeutic setting it affects the production of the effector Th2 cytokines. Further work is needed to determine the bacterial factors that are involved in the protective effect and whether BPZE1P can also protect against AAI in humans.

## Methods

### Bacterial strains and growth conditions

BPZE1 and BPZE1P were cultured at 37 °C on Bordet-Gengou (BG) agar (Difco) supplemented with 1% glycerol and 10% defibrinated sheep blood. For animal experiments, BPZE1 and BPZE1P were grown for 48 h on BG agar, then scraped off and resuspended in PBS. The density was adjusted to 10^6^ colony-forming units (CFU) per 20 µl (previously determined by CFU counts) to be administered intranasally.

### Mouse HDM sensitization and challenge

Groups of 4–5 week old female BALB/c mice were obtained from Charles River and maintained under specific pathogen-free (SPF) conditions at the Institut Pasteur de Lille animal facility. For infections mice were anaesthetized with a cocktail of ketamine, atropine and Valium administered intraperitoneally. Anaesthetized mice were vaccinated intranasally with 10^6^ CFU of BPZE1 or BPZE1P in 20 µl PBS, while other groups were left unvaccinated.

For HDM sensitization and challenge mice were anaesthetized and sensitized by intranasal introduction of HDM extract (from *Dermatophagoides farinae*) at an index of reactivity (IR) of 5 or 25 as indicated, in 40 µl (kindly provided by Stallergenes/Greer). At indicated time points following sensitization the mice were intranasally challenged with HDM at 5 IR in 40 µl once per day for 5 consecutive days. 48 h following the final challenge mice were anaesthetized and intratracheal intubation was performed for mechanical ventilation using a FlexiVent (SCIREQ) device during which mice were exposed to increasing concentrations of nebulised methacholine (from 0–50 mg/ml in PBS). The mean airway resistance value was measured and normalized to the baseline.

### Analysis of Ig levels

Blood was drawn from the aorta and serum was collected by centrifugation at 5,000 x *g* for 5 min and stored at −20 °C. Total IgE and HDM-specific IgG1 in sera were measured by ELISA. For total IgE, 96-well plates (Corning Incorporated) were coated with 2 µg/ml rat anti-mouse IgE (clone R35–72; BD Biosciences Pharmingen, Cat#553413; dilution 1/250). After blocking and incubation of the serum samples, plates were incubated with 2 µg/ml biotinylated anti-mouse IgE antibody (clone R35–118; BD Biosciences Pharmingen, Cat#553419; dilution 1/250), followed by streptavidin conjugated to horse radish peroxidase (Zymed, Invitrogen) and TMB substrate (Interchim). The IgE concentration was expressed as µg/ml by comparison with murine IgE standard (0.5 mg/ml, BD Biosciences Pharmingen). For HDM-specific IgG1, microplates were coated with 10 IR/ml of HDM extract. After blocking and incubation with serum in serial dilutions, the antibodies were detected by addition of horse radish peroxidase-conjugated goat anti-mouse-IgG1 (Southern Biotechnology, Birmingham, Cat#1070-05; dilution 1/30,000), followed by addition of TMB substrate. OD_450_ was measured on the ELISA plate reader FLUOSTAR (Omega).

### BAL cell infiltration counts

Bronchoalveolar lavage (BAL) fluid was collected from mice that had been sacrificed by introduction and removal of 1 ml PBS into the lungs via intratracheal intubation. Cells were recovered by centrifugation at 1200 rpm for 5 min at 4 °C and resuspended in PBS for counting. Cells were then spun onto slides (Shandon cytospin 4, Thermo Fisher Scientific) and stained with May Grünwald Giemsa (DiffQuik) for differentiation of cell types^43^. This stain contains a mixture of azure, methylene blue and eosin dye which differentiates eosinophils in pink, lymphocytes as small cells in dark blue with a very thin cytoplasm and macrophages as large cells with a large cytoplasm. At least 500 cells were counted for each slide.

### Cytokine concentrations in lung extracts

Following BAL collection, the right lung lobes were harvested and frozen in liquid nitrogen for protein extraction. Whole lung lobes were placed in 1 ml lysis buffer (PBS; 0.5% nonidet P40 and protease inhibitor cocktail, Roche) and homogenised at 4 °C using a T-18 Ultra-Turrax (Ika). The samples were centrifuged and supernatants were collected. Total protein concentration in the supernatant was measured using Pierce BCA protein assay (Thermo Fisher Scientific). Cytokine and chemokine measurements were performed using the Cytokine 20-plex Mouse Panel (Invitrogen, Thermo Fisher Scientific) or the Chemokine 1 and Cytokine 26-plex Mouse Procartaplex (Invitrogen) according to the manufacturer’s instructions. Cytokine and chemokine concentrations were normalized to the concentration of total protein. CCL17, CCL11 and IL-33 levels were measured using the Mouse CCL17 DuoSet ELISA kit (R&D Systems), the Mouse CCL11 DuoSet ELISA kit (R&D Systems) and the IL-33 Mouse Uncoated ELISA kit (Invitrogen), respectively.

### Histology

The left lobe of the lung from each mouse was fixed in Antigenfix (Diapath) and embedded in paraffin (Histowax, HistoLab) according to the manufacturer’s indications. Histology coloration staining was carried out on 5 µm thick slides using an automated protocol developed for the Tissue-Tek PRISMA automated slide stainer with special stain capability (Sakura, Inc.). Slides were stained by Periodic Acid Schiff (PAS-Alfa Aesar) staining. Histology microscopic analysis was performed with a Nikon Eclipse Ni microscope (Nikon). Photographs were taken with a LAS X digital camera (Microscope Leica DM3000 LED) and processed with Image J. Mucus in the airway epithelium was quantified based on a five-point system: 0, no mucus; 1, <25% of the epithelium; 2, 25–50% of the epithelium; 3, 50–75% of the epithelium; 4, >75% of the epithelium^44^.

### Statistical analyses

Two-way repeated measure analysis of variance with Bonferroni post-test was performed using GraphPad Prism software for analysis of airways resistance. Other data were analysed using Kruskal-Wallis with a Conover post-test using R.

### Ethics statement

All animal experiments were carried out according the guidelines of the French Ministry of Research on animal experiments and with institutional regulations and ethical guidelines (B59-350009; Institut Pasteur de Lille, Lille, France). The protocols were approved by the Ethical Committees of the Region Nord Pas de Calais and the Ministry of Research (agreement number APAFIS#9107_201603311654342 V3). Experiments were conducted by qualified, accredited personnel.

### Reporting Summary

Further information on research design is available in the Nature Research Reporting Summary linked to this article.

## Supplementary information

REPORTING SUMMARY

## Data Availability

Raw data are available upon reasonable request.
