# Supplementary material for: Live attenuated pertussis vaccine for prevention and treatment of allergic airway inflammation in mice
Source: NPJ Vaccines. 2022 Jun 23;7:66. doi: 10.1038/s41541-022-00494-w (PMC9226346; doi:10.1038/s41541-022-00494-w)
Supplement: Supplementary file 1 — REPORTING SUMMARY [file 41541_2022_494_MOESM1_ESM.pdf]

nature portfolio

Corresponding author(s): Dr Camille Locht

Last updated by author(s): Mar 28, 2022

Reporting Summary

Nature Portfolio wishes to improve the reproducibility of the work that we publish. This form provides structure for consistency and transparency in reporting. For further information on Nature Portfolio policies, see our Editorial Policies and the Editorial Policy Checklist.

Statistics

For all statistical analyses, confirm that the following items are present in the figure legend, table legend, main text, or Methods section.

n/a	Confirmed	
☐	☒	The exact sample size (n) for each experimental group/condition, given as a discrete number and unit of measurement
☐	☒	A statement on whether measurements were taken from distinct samples or whether the same sample was measured repeatedly
☐	☒	The statistical test(s) used AND whether they are one- or two-sided<br>*Only common tests should be described solely by name; describe more complex techniques in the Methods section.*
☒	☐	A description of all covariates tested
☒	☐	A description of any assumptions or corrections, such as tests of normality and adjustment for multiple comparisons
☐	☒	A full description of the statistical parameters including central tendency (e.g. means) or other basic estimates (e.g. regression coefficient) AND variation (e.g. standard deviation) or associated estimates of uncertainty (e.g. confidence intervals)
☐	☒	For null hypothesis testing, the test statistic (e.g. F, t, r) with confidence intervals, effect sizes, degrees of freedom and P value noted<br>*Give P values as exact values whenever suitable.*
☒	☐	For Bayesian analysis, information on the choice of priors and Markov chain Monte Carlo settings
☒	☐	For hierarchical and complex designs, identification of the appropriate level for tests and full reporting of outcomes
☒	☐	Estimates of effect sizes (e.g. Cohen's d, Pearson's r), indicating how they were calculated

Our web collection on statistics for biologists contains articles on many of the points above.

Software and code

Policy information about availability of computer code

Data collection	GraphPad Prism software
Data analysis	GraphPad Prism software<br>Kruskal-Wallis with a Conover post-test using R

For manuscripts utilizing custom algorithms or software that are central to the research but not yet described in published literature, software must be made available to editors and reviewers. We strongly encourage code deposition in a community repository (e.g. GitHub). See the Nature Portfolio guidelines for submitting code & software for further information.

Data

Policy information about availability of data

All manuscripts must include a data availability statement. This statement should provide the following information, where applicable:

- Accession codes, unique identifiers, or web links for publicly available datasets
- A description of any restrictions on data availability
- For clinical datasets or third party data, please ensure that the statement adheres to our policy

Raw data are available upon reasonable request

Field-specific reporting

Please select the one below that is the best fit for your research. If you are not sure, read the appropriate sections before making your selection.

☒ Life sciences ☐ Behavioural & social sciences ☐ Ecological, evolutionary & environmental sciences

For a reference copy of the document with all sections, see nature.com/documents/nr-reporting-summary-flat.pdf

Life sciences study design

All studies must disclose on these points even when the disclosure is negative.

Sample size	Sample sizes were chosen based on previous studies with the same model, to respect as much as possible the 3R criteria for animal experiments
Data exclusions	No data were excluded
Replication	Data are representatives of at least two independent experiments
Randomization	Mice were randomly assigned to each treatment group
Blinding	No blinding was applied for animal experiments

Reporting for specific materials, systems and methods

We require information from authors about some types of materials, experimental systems and methods used in many studies. Here, indicate whether each material, system or method listed is relevant to your study. If you are not sure if a list item applies to your research, read the appropriate section before selecting a response.

Materials & experimental systems

n/a	Involved in the study	
☐	☒	Antibodies
☒	☐	Eukaryotic cell lines
☒	☐	Palaeontology and archaeology
☐	☒	Animals and other organisms
☒	☐	Human research participants
☒	☐	Clinical data
☒	☐	Dual use research of concern

Methods

n/a	Involved in the study	
☒	☐	ChIP-seq
☒	☐	Flow cytometry
☒	☐	MRI-based neuroimaging

Antibodies

Antibodies used	rat anti-mouse IgE (clone R35-72; BD Biosciences Pharmingen); biotinylated anti-mouse IgE antibody (clone R35-118; BD Biosciences Pharmingen); murine IgE standard (0.5 mg/ml, BD Biosciences Pharmingen); horse radish peroxidase-conjugated goat anti-mouse-IgG1 (Southern Biotechnology, Birmingham); Cytokine 20-plex Mouse Panel (Invitrogen, Thermo Fisher Scientific); Chemokine 1 and Cytokine 26-plex Mouse Procartaplex (Invitrogen); Mouse CCL17 DuoSet ELISA kit (R&D Systems); Mouse CCL11 DuoSet ELISA kit (R&D Systems); IL-33 Mouse Uncoated ELISA kit (Invitrogen);
Validation	Antibodies and kits were validated by the supplier

Animals and other organisms

Policy information about studies involving animals; ARRIVE guidelines recommended for reporting animal research

Laboratory animals	4-5 week old female BALB/c
Wild animals	The study did not involve wild animals
Field-collected samples	The study did not involve samples collected from fields
Ethics oversight	All animal experiments were carried out according the guidelines of the French Ministry of Research on animal experiments and with institutional regulations and ethical guidelines (B59-350009; Institut Pasteur de Lille, Lille, France). The protocols were approved by the Ethical Committees of the Region Nord Pas de Calais and the Ministry of Research (agreement number APAFIS#9107_201603311654342 V3). Experiments were conducted by qualified, accredited personnel.

Note that full information on the approval of the study protocol must also be provided in the manuscript.
